# Acoustic characteristics used by Japanese macaques for individual discrimination

**DOI:** 10.1242/jeb.154765

**Published:** 2017-10-01

**Authors:** Takafumi Furuyama, Kohta I. Kobayasi, Hiroshi Riquimaroux

**Affiliations:** Graduate School of Life and Medical Sciences, Doshisha University, Kyoto, Japan

**Keywords:** Go/no-go operant conditioning, STRAIGHT, Fundamental frequency, Vocal tract characteristics

## Abstract

The vocalizations of primates contain information about speaker individuality. Many primates, including humans, are able to distinguish conspecifics based solely on vocalizations. The purpose of this study was to investigate the acoustic characteristics used by Japanese macaques in individual vocal discrimination. Furthermore, we tested human subjects using monkey vocalizations to evaluate species specificity with respect to such discriminations. Two monkeys and five humans were trained to discriminate the coo calls of two unfamiliar monkeys. We created a stimulus continuum between the vocalizations of the two monkeys as a set of probe stimuli (whole morph). We also created two sets of continua in which only one acoustic parameter, fundamental frequency (*f*_0_) or vocal tract characteristic (VTC), was changed from the coo call of one monkey to that of another while the other acoustic feature remained the same (*f*_0_ morph and VTC morph, respectively). According to the results, the reaction times both of monkeys and humans were correlated with the morph proportion under the whole morph and *f*_0_ morph conditions. The reaction time to the VTC morph was correlated with the morph proportion in both monkeys, whereas the reaction time in humans, on average, was not correlated with morph proportion. Japanese monkeys relied more consistently on VTC than did humans for discriminating monkey vocalizations. Our results support the idea that the auditory system of primates is specialized for processing conspecific vocalizations and suggest that VTC is a significant acoustic feature used by Japanese macaques to discriminate conspecific vocalizations.

## Abstract (precis)

**Summary:** The acoustic features used by Japanese macaques to discriminate among individuals was investigated. Monkeys used both fundamental frequencies and vocal tract characteristics to discriminate between two monkeys based on vocalizations.

## INTRODUCTION

Most primates, including humans, can distinguish the voices of different conspecifics. Previous studies have shown that common squirrel monkey (*Saimiri sciureus*; Kaplan et al., 1978), vervet monkey (*Chlorocebus pygerythrus*; Cheney and Seyfarth, 1980), Japanese monkey (*Macaca fuscata*; Pereira, 1986) and rhesus macaque (*Macaca mulatta*; Jovanovic et al., 2000) mothers could distinguish the voices of their own infants from those of other juvenile individuals. Pygmy marmosets discriminated vocalizations of other group members (Snowdon and Cleveland, 1980). Another study showed that rhesus macaques were able to discriminate species-specific vocalizations of kin from those of non-kin (Rendall et al., 1996). Taken together, these studies indicate that the identification of individuals by their vocalizations is important in many primates.

The acoustic characteristics used by primates to discriminate conspecific individuals have been investigated. Owren et al. (1997) analyzed the vocalizations of female chacma baboons (*Papio ursinus*) and reported that the acoustic features of vocal tract filtering may reflect individuality. Bachorowski and Owren (1999) analyzed phonemes in the speech of humans and showed that vocal tract filtering may contribute to the identification of individuals. The resonance of vocal tract characteristics (VTC) may affect individual identification in rhesus macaques (Rendall et al., 1998), lemurs (Gamba et al., 2012) and Japanese macaques (Furuyama et al., 2016). Statistical analyses have shown that the acoustic features of the fundamental frequency (*f*_0_), such as the beginning frequency and the maximum frequency, in addition to the formants, can be a reliable cue for identifying callers in several monkey species (Smith et al., 1982; Snowdon et al., 1983).

The species-specific communication sound, called the ‘coo call’, of Japanese macaques was used in the present study. Green (1975) classified the vocalizations of Japanese macaques in the field and showed that monkeys possessed several types of call. Since then, many other research groups have also focused on their vocalization behavior. Coo calls have both a clear *f*_0_ and rich harmonics, and the calls are important for social interactions. Monkeys vocalize coo calls when they approach other individuals closely for grooming (Mori, 1975). Brown et al. (1979) showed that monkeys could distinguish sound localizations using coo calls. The monkeys exchange coo calls with each other (Mitani, 1986), and match the *f*_0_ of their reply with that of the preceding calls (Sugiura, 1998). Japanese macaques vocalize greeting calls (coo calls, grunts and girneys) together with increased social interactions when they approach unrelated females (Katsu et al., 2014). In a previous study, we investigated the acoustic features used for individual discrimination using synthetic coo calls that had the same *f*_0_ (Furuyama et al., 2016). In natural observations, however, the *f*_0_ varied among individuals when they uttered spontaneous vocalizations (Mitani, 1986; Sugiura, 1998). In the present study, we investigated the acoustic features (*f*_0_ and VTC) used by Japanese macaques and human subjects for discriminating monkey vocalizations with differing *f*_0_ and VTC. We used standard go/no-go operant conditioning and speech processing techniques to systematically compare the perceptual contributions of different acoustic features of monkey vocalizations.

## MATERIALS AND METHODS

### Subjects

Two male Japanese macaques (monkeys 1 and 2; 7 and 10 years old, respectively, at the time of testing) and five male humans (22–23 years old) participated in these experiments. Each monkey was housed individually in a primate cage under a constant 13 h:11 h light:dark cycle. Access to liquids was limited because water served as a positive reinforcement in the experiments. All experiments were conducted in accordance with guidelines approved by the Animal Experimental Committee of Doshisha University, Japan and the Ethics Board of Doshisha University.

### Apparatus

All training and tests were conducted in a sound-attenuated room (length×width×height: 1.70×1.85×2.65 m). In the experiments involving the monkeys, the subjects were seated in a monkey chair equipped with a drinking tube and a response lever. In the experiments involving human subjects, the same lever was attached to a desk, and the subject was seated in a standard laboratory chair in front of the desk. A loudspeaker (SX-WD1KT; Victor, Tokyo, Japan) driven by an amplifier (SRP-P2400; Sony, Tokyo, Japan) was positioned 58 cm in front of the subject's head at the same height as the ears. The frequency response of the speaker characteristics was flattened (±3 dB) between 0.4 and 16 kHz using a graphic equalizer (GQ2015A; Yamaha, Hamamatsu, Japan). A white-light-emitting diode (LED) and a charge-coupled device (CCD) video camera were attached to the top of the speaker. The LED was lit during the training and test sessions for lighting, and subjects were monitored using the CCD camera.

### Acoustic stimuli

Coo calls from two monkeys that were not familiar to monkeys 1 and 2 [monkey A (cooA) and monkey B (cooB)] were recorded as sound stimuli in a sound-attenuated room (1.70×1.85×2.65 m) using a digital audio tape recorder (TCD-D8; Sony) and a condenser microphone [range of frequency response (±3 dB): 3–20,000 Hz; type 7046, Aco, Tokyo, Japan] at a sampling rate of 44.1 kHz and a resolution of 16 bits. The subjects (both monkeys and humans) did not hear the voices of the stimulus monkeys prior to the experiment. Fourteen coo calls (seven from each monkey) with signal-to-noise ratios greater than 40 dB were selected randomly from the recorded sounds for use as stimuli.

**Fig. 1.**
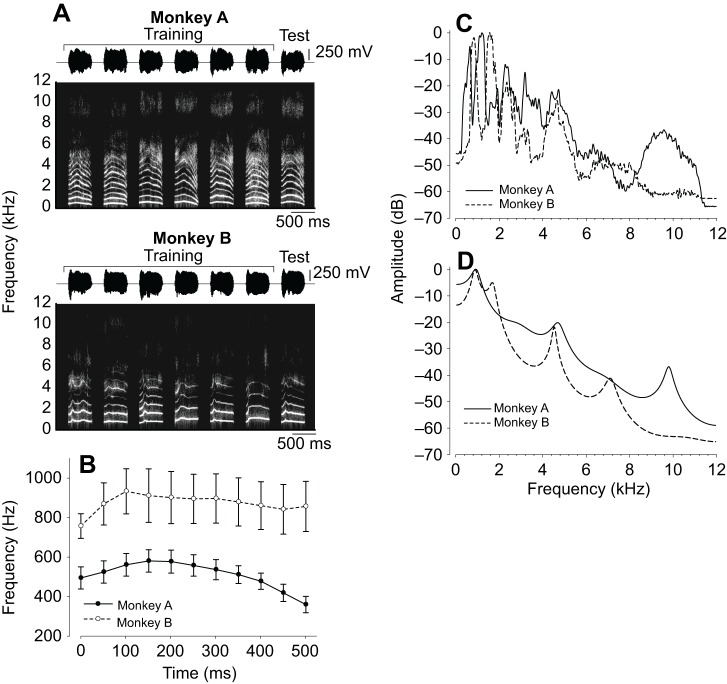
**Acoustic features of coo calls.** (A) Coo calls for monkey A (cooA) and monkey B (cooB). For each monkey, the waveform envelope is shown above the spectrogram. The recorded calls were changed such that they had the same durations and amplitude envelopes. The right-most calls were used to synthesize the test stimuli. These spectrograms were based on 512-point fast Fourier transform (FFT; Hamming window) with 50% overlap. (B) Mean temporal pitch patterns of the coo calls of the two monkeys (cooA and cooB). Error bars: s.d. (C) Power spectra of cooA (solid line) and cooB (dashed line) stimuli. (D) Linear predictive coding spectra of cooA (solid line) and cooB (dashed line). The data illustrate the differences in the vocal tract characteristics (VTC) of the two monkeys.

**Fig. 2.**
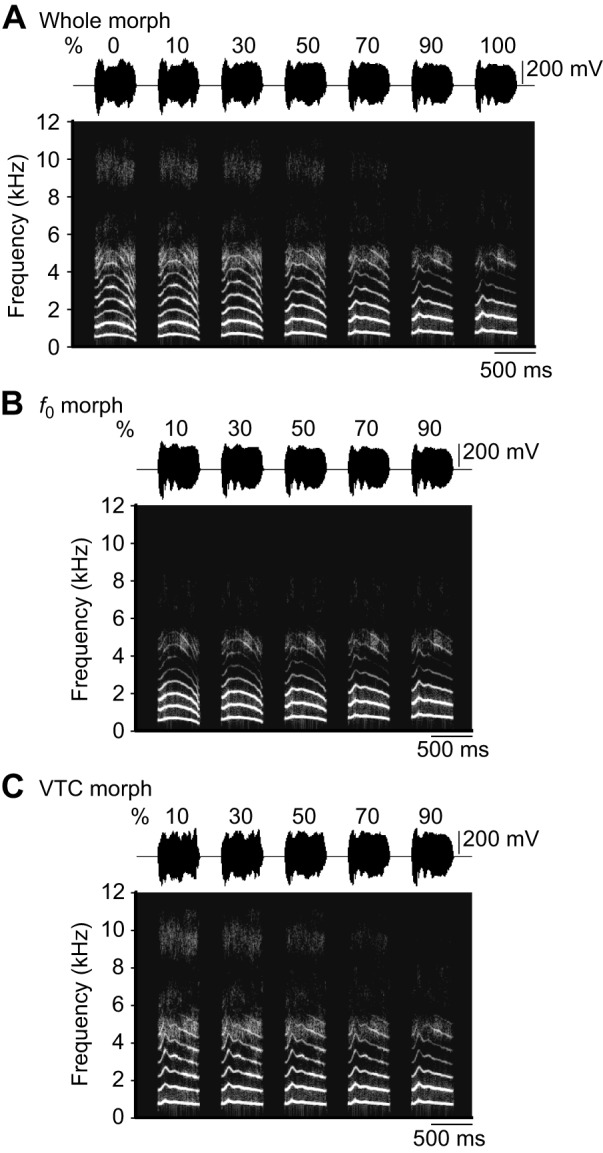
**Spectrograms of stimulus continua between the coo calls of two monkeys.** Numbers above the spectrograms represent the percentage of cooB. (A) Whole morph. (B) *f*_0_ morph. (C) VTC morph. In B and C, the designated acoustic feature was morphed between cooA and cooB, whereas the other feature remained constant (i.e. as cooB).

Recorded coo calls (Fig. 1A) were analyzed using a digital-signal-processing package (STRAIGHT; Kawahara et al., 1999) to measure three acoustic parameters: the *f*_0_ (Fig. 1B), VTC (the frequency structure corresponding primarily to the resonance characteristics of the vocal tract; Fig. 1C,D) and the durations of the coo calls. Twelve coo calls (six per individual) of the total of fourteen were used as training stimuli (cooAs and cooBs). One coo call from each monkey (cooA and cooB) was not played during training and was used to synthesize a test stimulus. Three continuum stimuli of coo calls were created using STRAIGHT. The program was used to break down a coo call into two acoustic parameters (*f*_0_ and VTC), which allowed us to manipulate the parameters independently. For example, we might synthesize a coo call from 30% of the information from monkey A (i.e. cooA) and 70% of the information from monkey B (i.e. cooB) into one acoustic parameter (e.g. *f*_0_) while using no information from monkey A in another parameter (e.g. VTC). A stimulus continuum, defined as a whole morph, consisting of cooA and cooB, was created to comprise 10, 30, 50, 70 and 90% of cooB (Fig. 2A, Audio 1). Each stimulus in the continuum contained equal contributions from the *f*_0_ and VTC from cooB. We created two additional sets of stimulus continua in which only one acoustic parameter, *f*_0_ or VTC, was changed from cooA to cooB, whereas the other acoustic feature remained the same as monkey B's original. One stimulus continuum, defined as the *f*_0_ morph, was created to comprise 10, 30, 50, 70 and 90% of the *f*_0_ from cooB (Fig. 2B, Audio 2); the other, defined as the VTC morph, comprised 10, 30, 50, 70 and 90% of the VTC from monkey B (Fig. 2C, Audio 3). Three different sound pressure level (SPL) stimuli were created for each stimulus type: 57, 60 and 63 dB SPL (re. 20 μPa). All stimulus amplitudes were modified digitally and calibrated (using a microphone; type 7016, Aco). The call durations were equalized to 517 ms (the average of all calls) via linear time-stretching or -compressing using STRAIGHT.

### Procedure

**Fig. 3.**
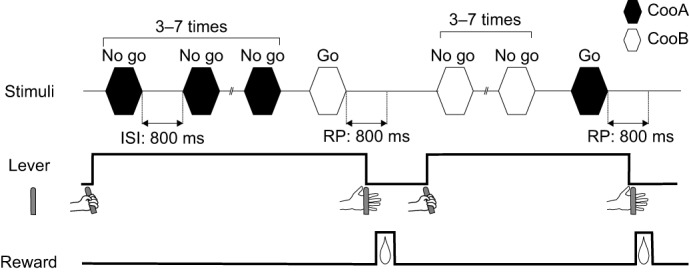
**Schematized trial event sequence.** Top: timing of the stimulus. Middle: response of the animal. Bottom: timing of the reward. Black hexagon: cooA; white hexagon: cooB. The subjects were required to press a lever to begin the trial. Then, cooA was presented three to seven times with an interstimulus interval of 800 ms. The subjects were required to continue pressing the lever while cooA was repeated. When cooB (go stimulus) was presented, the subjects were required to release the lever within 800 ms after the offset of cooB to receive a reward. After a correct response to a go stimulus, the stimulus contingencies were reversed in the next trial. That is, cooA became the go stimulus, and cooB became the no-go stimulus. In the test trial, a test stimulus was presented after cooB, repeated three to seven times. Neither a reward nor a punishment followed a test trial. ISI, interstimulus interval; RP, response period.

Standard go/no-go operant conditioning was used. Fig. 3 shows the schematized event sequence of the trials. Subjects were required to press the lever switch on the monkey chair to begin the trial. Then, coo calls from the same subject, monkey A or monkey B, were presented randomly three to seven times. In the repetition, call types were selected randomly from 18 types of stimulus (one individual×six types of coo calls×three intensities). The interstimulus interval between adjacent stimuli was 800 ms. While the calls from the same monkey were presented (no-go trial), subjects were required to continue pressing the lever. When the stimulus was changed from one monkey to another (go trial), subjects were required to release the lever within 800 ms from the offset of the stimulus. For example, a trial was started using the repeated playback of cooAs (no-go stimulus). In the repetition, the cooA type (out of six) and the stimulus intensity (out of three: 57, 60 and 63 dB SPL) were changed randomly. The subjects were required to continue pressing the lever while cooA was repeated [correct rejection (CR)]. When cooB (go stimulus) was presented, the subjects were required to release the lever within 800 ms after the offset of cooB (hit). Hits were reinforced by fruit juice (2 ml). When the subjects released the lever during the repetition period of the no-go stimulus (false alarm) or failed to release the lever within 800 ms after the go stimulus (miss), a 5- to 10-s timeout period accompanied by turning off the LED was provided as feedback. When the subjects responded successfully to the go stimulus, the stimulus contingencies were reversed in the next trial. That is, the next trial was started using a playback of cooB instead of cooA, and the subject had to release the lever when cooA was played to receive the reward.

Performance was measured by the correct response percentage (CRP; total percentage of hits and CRs). In total, 130–180 go trials (trials in which the stimulus changed from one monkey to the other) and 800–1000 no-go trials were presented per day to both subjects.

After the monkey's scores exceeded the CRP threshold (75%), the subject proceeded to the test session. Test trials were conducted approximately every 10–20 training trials. A test stimulus was presented after cooB, repeated three to seven times, and each type of test stimulus was played six times. Neither a reward nor a punishment followed a test trial.

For the human subjects, no juice was given as a reward in the trials, and a CRP of 90% was used as the threshold for proceeding to the test session. Test trials were conducted every five to ten training trials, and each type of test stimulus was presented five times.

### Data analysis

We measured the go response rates and reaction times between the end of each stimulus and the release of the lever switch. The *d*′ sensitivity values were calculated from the signal detection theory (Green and Swets, 1966) by subtracting *z*-score (normal deviates) of ‘false alarm’ rates from *z*-score of ‘hit’ rates. The coefficient of correlation (Spearman's rank order correlation coefficient) between reaction times and sets of continuum stimuli were calculated using commercial statistics software (SPSS; IBM, NY, USA).

## RESULTS

### Training results in each subject

Monkeys 1 and 2 required 20 and 21 days of training, respectively, to distinguish between the sets of cooA and cooB. Two days before the test day, the monkeys scored CRPs of 85% (monkey 1: *d*′=1.81) and 91% (monkey 2: *d*′=2.48). One day before the test day, the CRPs were 85% (monkey 1: *d*′=1.89) and 86% (monkey 2: *d*′=2.09). The CRPs for all human subjects were >90% during the training sessions. During the test period, the CRPs to training stimuli were >75% in both monkeys [monkey 1: 86% (*d*′=1.82); monkey 2: 87% (*d*′=2.09)] and >90% in all humans [human 1: 98% (*d*′=4.26); human 2: 99% (*d*′=4.78); human 3: 99% (*d*′=5.29); human 4: 98% (*d*′=4.14); human 5: 99% (*d*′=4.38)]. The CRPs during the test period did not differ from those during the training sessions, and the corresponding *d*′ sensitivity values remained higher than 1.8. The *d*′ sensitivity value was comparable to several previous studies using rhesus monkeys (e.g. Hage and Nieder, 2013). These results indicate that the subjects maintained the same discriminatory performance they showed in response to the training stimuli throughout the experiment. In addition, we did not find any significant correlation between CRP and the number of no-go stimuli repeated before the go stimulus (monkey 1: *r*=0.39, *P*=0.36; monkey 2: *r*=0.24, *P*=0.59).

### Morphed stimuli between cooA and cooB: whole morph

**Fig. 4.**
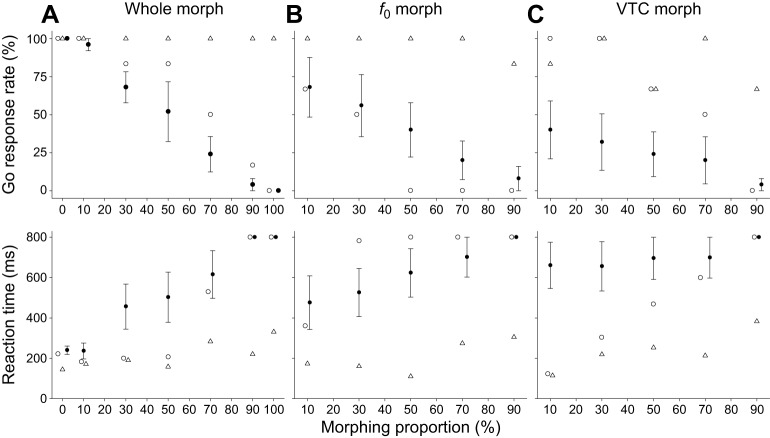
**Go response rates and reaction times to the test stimuli.** White circle: monkey 1; white triangle: monkey 2; black circle: mean for humans. Error bars: s.e.m. The abscissa represents the proportion of cooB (no-go stimulus). (A) Responses to whole-morph stimuli. (B) Responses to *f*_0_-morph stimuli. (C) Responses to VTC-morph stimuli.

The go response rates to the whole-morph stimulus continuum (whole morph) are shown in Fig. 4A (top panel). The go response rates of monkey 1 and humans decreased gradually as a function of the increasing morph proportion of test cooB, but that of monkey 2 did not decrease. The go response rate of monkey 1 decreased to <50% when the morphing proportions increased to >70%. In humans, the average go response rates decreased to <50% when the morphing proportions increased to >50%.

Fig. 4A (bottom panel) shows the reaction times to the whole morph. The reaction times of both monkeys and humans increased gradually with the increase in the morphing proportion (Table 1). A significant positive correlation was observed between the morphing proportions of cooB and the reaction times to the stimuli in both monkeys and humans (Spearman’s correlation coefficients, monkey 1: *r*_s_=0.62, *n*=42, *P*<0.001; monkey 2: *r*_s_=0.55, *n*=42, *P*<0.001; humans: *r*_s_=0.78, *n*=35, *P*<0.001). Both monkeys and humans pressed the lever longer as the stimulus became more similar to test cooB.

**Table 1.**
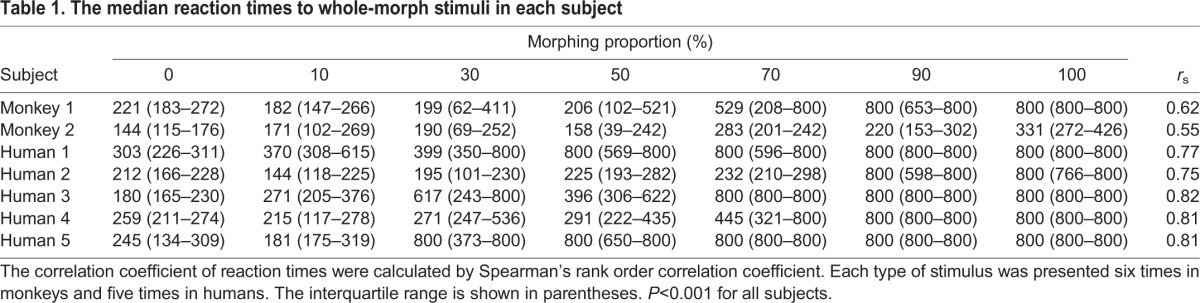
**The median reaction times to whole-morph stimuli in each subject**

### Morphed *f*_0_ continuum results

The go response rates of monkey 1 and humans decreased gradually with the increase in the morphing proportion of the *f*_0_ from test cooB, but that of monkey 2 did not decrease (Fig. 4B). The go response rates of monkey 1 decreased to <50% when the morphing proportion of the *f*_0_ from test cooB increased to >30%. In humans, the go response rates decreased to <50% when the morphing proportions increased to >50%.

The reaction times to the *f*_0_ morph are depicted in Fig. 4B and Table 2. The reaction times for subjects (in the two monkeys and the humans on average) increased as the proportion of the *f*_0_ from test cooB increased (monkey 1: *r*_s_=0.50, *n*=30, *P*=0.005; monkey 2: *r*_s_=0.46, *n*=30, *P*=0.01; humans: *r*_s_=0.48, *n*=25, *P*=0.015). The reaction times of three humans correlated with the morphing rates of *f*_0_ morphs (human 1: *r*_s_=0.80, *n*=25, *P*<0.001; human 2: *r*_s_=0.54, *n*=25, *P*=0.005; human 4: *r*_s_=0.71, *n*=25, *P*<0.001; Table 2). Both monkeys and humans pressed the lever longer as the *f*_0_-morph stimuli became more similar to test cooB.

**Table 2.**
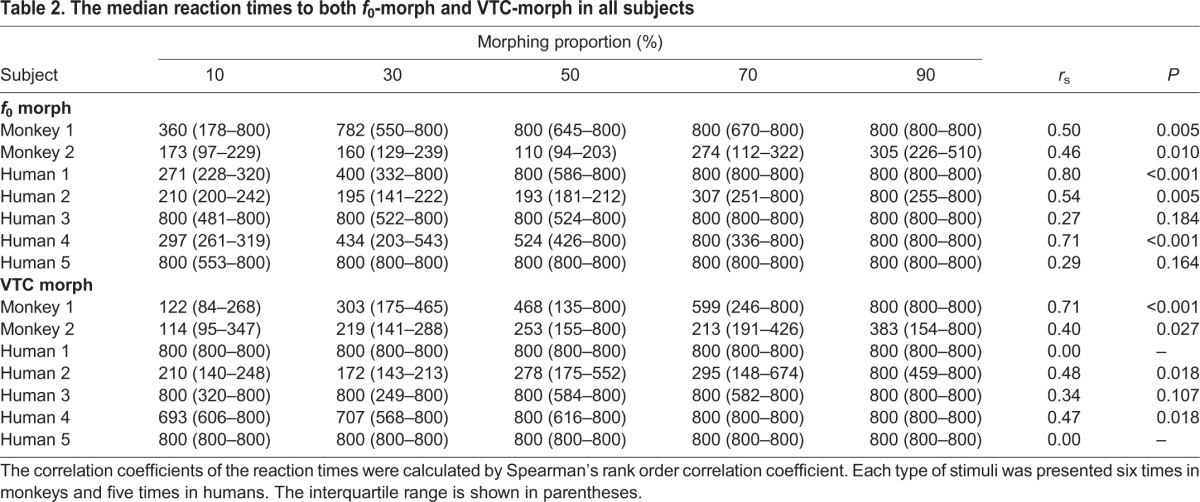
**The median reaction times to both *f*_0_-morph and VTC-morph in all subjects**

### Morphed VTC continuum results

The go response rate of monkey 1 decreased with the increase in the morphing proportion of the VTC from test cooB, whereas that of monkey 2 did not decrease systematically and remained >50% (Fig. 4C). For monkey 1, the go response rate decreased to <50% when the morphing proportions of the VTC of test cooB increased to >70%. In humans, the go response rates remained <50% regardless of the morphing proportion in the VTC morph.

The reaction times to the VTC morph are depicted in Fig. 4C and Table 2. The reaction times of both monkeys increased significantly as the contribution of test cooB to the VTC increased (monkey 1: *r*_s_=0.71, *n*=30, *P*<0.001; monkey 2: *r*_s_=0.40, *n*=30, *P*<0.027). By contrast, on average, the median reaction time in humans did not correlate significantly with the morphing rates of VTC morphs (humans: *r*_s_=0.33, *n*=25, *P*=0.11) and remained constant over the VTC morph continuum. However, the reaction times of two humans correlated with the morphing rates of VTC morphs (human 2: *r*_s_=0.48, *n*=25, *P*=0.018; human 4: *r*_s_=0.47, *n*=25, *P*=0.018; Table 2), whereas their correlation coefficients for the *f*_0_ morph were higher than those of VTC morph (Table 2).

**Fig. 5.**
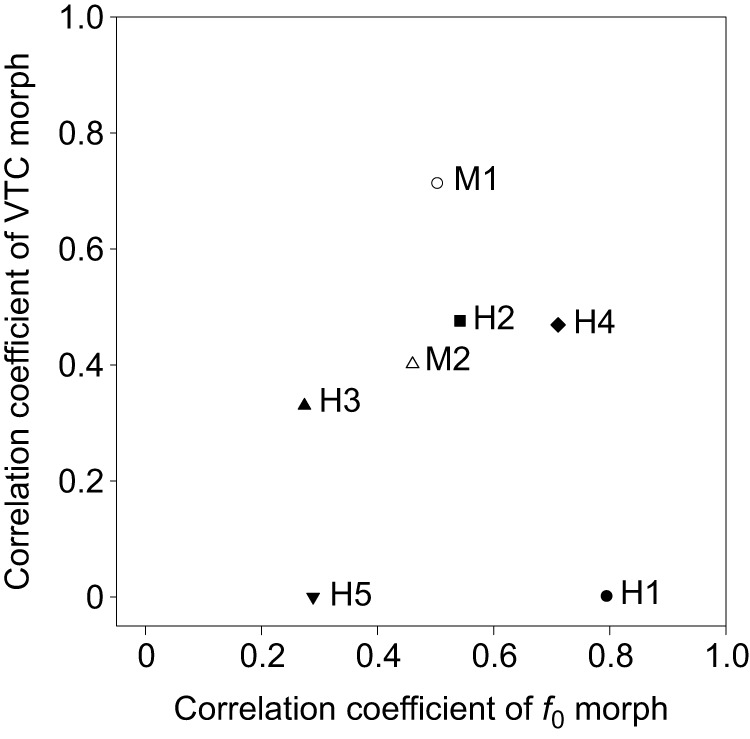
**Distribution of correlation coefficients for *f*_0_-morph and VTC-morph stimuli in each subject.** M: monkey; H: human. Correlation coefficients of reaction times to *f*_0_-morph and VTC-morph stimuli were calculated using Spearman's rank order correlation coefficient.

 Fig. 5 shows the distributions of correlation coefficients in the *f*_0_ and VTC morphs. To evaluate the species difference, we plotted the correlation coefficients for *f*_0_ and VTC morphs in Fig. 5. The ranges of correlation coefficients for the *f*_0_ morphs were 0.46–0.50 in monkeys and 0.27–0.80 in humans. The range of correlation coefficients for the VTC morph were 0.40–0.71 in monkeys and 0.00–0.48 in humans.

## DISCUSSION

With all continuum stimuli, the go response rates for monkey 1 and humans decreased with the increase in the morph proportion, whereas monkey 2's go response rates to the test stimuli remained relatively high (>50%; Fig. 4). Although both monkeys went through the same training regime, they seemed to use a different behavioral strategy. Monkey 1 released the lever (go response) less often as the probe stimulus more highly resembled the no-go stimulus (Fig. 4A), suggesting that it adjusted the go response rate according to the perceptual similarity between the test stimuli and learned stimulus set. By contrast, monkey 2 almost always released the lever to any probe stimulus, including test cooB (i.e. 100% whole morph), suggesting that it was able to discriminate test cooB from trained cooBs. In other words, test cooB and trained cooBs were perceptually different enough to evoke go responses for monkey 2. The *d*′ analysis showed that monkey 2 exhibited better discrimination than monkey 1; the perceptual difference could partially reflect the response difference to test cooB. Both monkeys, however, showed longer reaction times as the probe stimulus resembled the test cooB (Fig. 4A), indicating that call perception systematically changed along the stimulus continuum. Taken together, our data, except for the go response rate of monkey 2, suggest that subjects responded to the high morph proportion more similarly to how they responded to cooB than to cooA.

Several studies have indicated that the vocalizations of monkeys can be modified using STRAIGHT. Previously, the vocal tract lengths of rhesus monkeys were increased or decreased virtually using this software (Ghazanfar et al., 2007). Chakladar et al. (2008) demonstrated that the vocalizations of different macaque individuals could be morphed using this software, and the quality of the morphs was evaluated by human listeners. In the present study, the time taken by both monkeys and humans to release the lever increased gradually with an increase in the morph proportion (Fig. 4). To our knowledge, this is the first report of a stimulus continuum synthesized with STRAIGHT that was applied to monkey subjects and demonstrated that the stimuli systematically affected the perception of individuals.

The stimulus continuum has been used to investigate the detailed nature of perception and has been especially valuable in evaluating categorical perceptions (Kuhl and Padden, 1983; Miyawaki et al., 1975; Sinnott et al., 1976; Sinnott and Adams, 1987; Sinnott and Brown, 1997). A common feature in categorical perception is that the subject is more sensitive to a physical transition between two perceptual categories than to the same change occurring within a category. This has typically been measured using a combination of both a discrimination task involving adjacent stimulus pairs (e.g. 10 versus 30% morphed) in a stimulus continuum and an identification task along the continuum. Using our stimulus scheme, future research can examine how monkeys categorize vocalizations of different conspecifics.

We investigated the acoustic features used for individual discrimination using continuum stimuli. The reaction times of both monkeys and humans increased gradually with the increase in the morphing proportion involving the *f*_0_ morph (Fig. 4B), suggesting that, on average, both monkeys and humans used the *f*_0_ as a discriminative stimulus.

Several monkey species have been shown to discriminate vocalizations using the *f*_0_ in natural and experimental settings. This is consistent with the present results. The temporal structures of the *f*_0_ of Japanese macaques’ coo calls have been regarded as having behavioral significance because the monkeys modify the temporal structure depending on the situation (Green, 1975). Trained Japanese macaques showed an ability to discriminate the peak positions of natural tonal vocalizations (Zoloth et al., 1979). Monkeys may categorically discriminate the temporal structures of the *f*_0_ (May et al., 1989). Additionally, monkeys distinguish the synthetic vocalizations of conspecifics using the peak positions of the *f*_0_ (Hopp et al., 1992). In related research, Japanese macaques were trained to discriminate the vocalizations of different monkeys, and the subjects responded to the *f*_0_ as a discriminant stimulus for the task, suggesting that the *f*_0_ contributed to individual discrimination (Ceugniet and Izumi, 2004). Overall, the *f*_0_ plays a significant role in the vocal communication of many species of monkeys, including Japanese macaques. Our monkeys also used the *f*_0_ in our experimental setting.

In our stimulus set, the mean frequencies of cooB were higher, by about 350 Hz or 68%, than those of cooA [cooA: 519±50 Hz (mean±s.d.), cooB: 875±121 Hz; Fig. 1B]; the sensitivities of difference limits for frequency in monkeys and humans have been reported to be 14–33 Hz and 2.4–4.8 Hz, respectively (Prosen et al., 1990; Sinnott et al., 1985), suggesting that the average *f*_0_ alone can readily serve as a discriminative stimulus for both species. Additionally, the *f*_0_ of the cooA peak was earlier than that of the cooB peak by ∼60 ms (the peak position of the vocalizations: 95±22 ms for monkey A and 134±45 ms for monkey B). Japanese macaques and humans have shown the ability to distinguish changes in the peak position as small as 20–50 ms (Hopp et al., 1992), indicating that the temporal structure of the *f*_0_ can also function as a discriminative stimulus in both species. Thus, the *f*_0_ was such that both monkeys and humans could use it as a key to distinguish the stimulus sets.

Both monkeys took significantly longer to respond as the morphing proportion of the VTC morph increased (Fig. 4C). The results showed that monkeys used the formant frequencies, in addition to the *f*_0_, as discriminative stimuli for the stimulus sets. It has been shown that formants are biologically significant for the vocal communication of many primate species. In human speech, vocal tract length is necessary to classify individual speakers (Bachorowski and Owren, 1999). The resonances of the vocal tract have physical characteristics in baboons (Owren et al., 1997; Rendall, 2003). Owren (1990) showed that formants were used to distinguish alarm calls in a manner similar to that used by humans for discriminating speech. Similar to humans, trained Japanese macaques show great sensitivity to different formant frequencies (Sommers et al., 1992). Non-human primates are able to discriminate formant changes in species-specific vocalizations (Fitch and Fritz, 2006). One study using a preferential looking paradigm with non-trained monkeys showed that the index characteristics of age-related size were embedded in the formants of monkeys (Ghazanfar et al., 2007). Taken together, these results were consistent with the present results, as formant information played an important role in vocal communication, and the monkeys used the information to discriminate the stimulus sets.

In contrast, human behavioral data showed that the mean reaction times and go response rates did not change systematically as the morphing proportion of the VTC morph increased (Fig. 4C). These results indicated that, unlike the monkeys, the humans, on average, did not use the formant frequency as a key to discriminate the stimulus sets. This difference might stem from differences in auditory sensitivity. Japanese macaques have better high-frequency (i.e. >8 kHz) hearing than do humans (Heffner, 2004). The power spectrum peak at 10 kHz of cooA, the most distinct feature differentiating the stimulus sets (Fig. 1), could be more salient to monkeys than to humans. Thus, the VTC had a greater effect on the monkeys than on the humans.

Another explanation, which does not necessarily contradict that of auditory sensitivity, involves a difference in auditory processing. Previous studies have shown that humans are more sensitive than are monkeys to the discrimination of formant transitions, although monkeys are able to distinguish linguistic sounds (Sinnott et al., 1976; Sinnott and Brown, 1997). Another study compared differences in the sensitivity of humans and monkeys using a continuum of voice onset time (VOT) in English; it was suggested that the sensitivity with which pairs of syllables can be discriminated in VOT was less in monkeys than in humans (Sinnott and Adams, 1987). Our behavioral data indicated that the auditory system of monkeys is specialized to process their vocalizations, especially the biologically significant acoustic cue of their VTC.

Our previous study used synthetic coo calls to evaluate the acoustic features used for individual recognition, and the mean *f*_0_ of the vocalization stimulus was equalized to the same frequency, whereas the temporal modulation of the *f*_0_ was unchanged. The results suggested that VTC is more important than the temporal structures of the *f*_0_ for discriminating individuals (Furuyama et al., 2016). In field studies, however, the *f*_0_ of the spontaneous vocalizations of Japanese monkeys differed by 300–1000 Hz between individuals (Mitani, 1986; Sugiura, 1998). In the present study, we used stimulus sets that differed in *f*_0_ by ∼350 Hz (cooA: 519±50 Hz; cooB: 875±121 Hz; Fig. 1B), which is comparable to natural individual differences. The current results, together with those of our previous study, suggest that monkeys used both the *f*_0_ and VTC to discriminate individuals and that they can adjust their reliance on the *f*_0_ depending on the stimulus.

The distributions of correlation coefficients differed between monkeys and humans (Fig. 5), but the correlation coefficients of the two monkeys were similarly distributed. Thus, the monkeys used the *f*_0_ and VTC to discriminate the vocalizations of a monkey in a relatively similar way. By contrast, the distributions of correlation coefficients differed among the human subjects. For example, two humans (humans 1 and 5) used only the *f*_0_, whereas three humans (humans 2, 3 and 4) used both the *f*_0_ and VTC to distinguish the caller monkey. Interestingly, the reaction times of human 2 in both the *f*_0_ morph and VTC morph were similar to those of monkeys rather than those of other human subjects (Fig. 4 bottom, and Fig. 5), suggesting that some humans are capable of discriminating the monkey vocalization as monkeys do. As a whole, each human may have used a different strategy in utilizing acoustic cues (i.e. *f*_0_ or VTC) to discriminate the monkey vocalization.

We investigated only males as human subjects in this experiment. As our results show, males on average did not depend on formant frequencies to discriminate monkey vocalizations. Previous study of humans showed that males were more sensitive than females in distinguishing acoustic size by using formant frequencies of synthesized human voices (Charlton et al., 2013). If this sex difference holds in perception of heterospecific vocalizations (i.e. monkey voice), the human (average of female and male) might rely on formant frequencies even less in discriminating monkey vocalizations than present data imply. However, further study is needed to obtain a more complete picture of the species difference.

This species difference in how strongly and consistently they relied on VTC for the purpose of discrimination might stem from differences in auditory processes. Many neurophysiological studies have shown that primates develop brain mechanisms specialized for processing conspecific vocalizations. In studies with non-human primates, neurons in the auditory cortex also responded to species-specific vocalizations rather than to both non-vocal stimuli (Winter and Funkenstein, 1973) and synthetic vocalizations that were spectro-temporal structures changed based on natural vocalizations (Wang et al., 1995). Another study showed that the left temporal cortex, including the auditory cortex, was necessary for Japanese macaques to discriminate different types of conspecific vocalizations (Heffner and Heffner, 1984). It has also been shown that interhemispheric interactions create a conspecific-vocalization-specific response in the left hemisphere (Poremba et al., 2004). A recent study demonstrated that the brain activities in the superior temporal plane responded selectively not only to species-specific vocalizations but also to the identity of conspecific individuals (Petkov et al., 2008). Our results reinforce the idea that primates have a cognitive faculty for processing conspecific vocalizations, and suggest that VTC could be one of the most important acoustic features that the monkey auditory system has to deal with.

## Supplementary Material

Supplementary information
