# Supplementary material for: Acoustic characteristics used by Japanese macaques for individual discrimination
Source: J Exp Biol. 2017 Oct 1;220(19):3571–8. doi: 10.1242/jeb.154765 (PMC5665434; doi:10.1242/jeb.154765)
Supplement: Supplementary information [file jexbio-220-154765-s1.pdf]

Supplemental Information

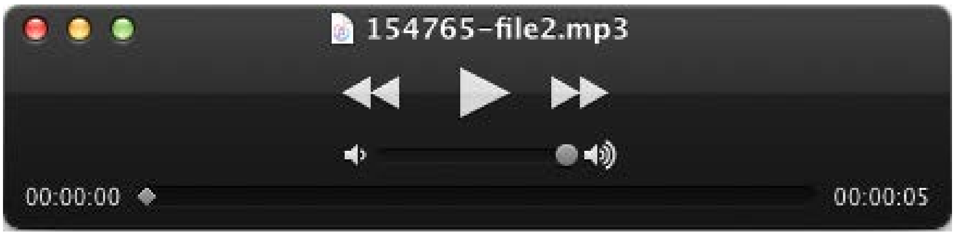

Supplemental Audio 1: Whole morph. Continuum stimuli between Coo calls of monkey A (cooA) and monkey B (cooB) used in the experiment.

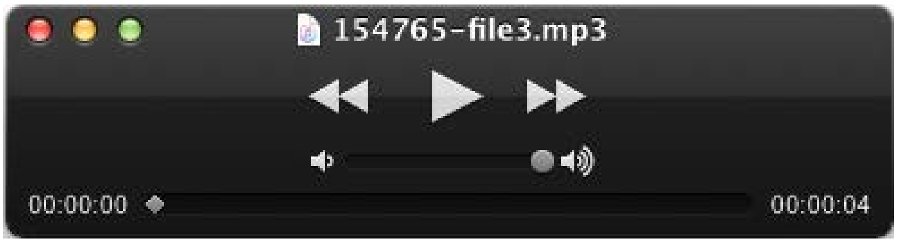

Supplemental Audio 2: f_0 morph. Continuum stimuli between fundamental frequencies (f_0) of monkey A and monkey B used in the experiment.

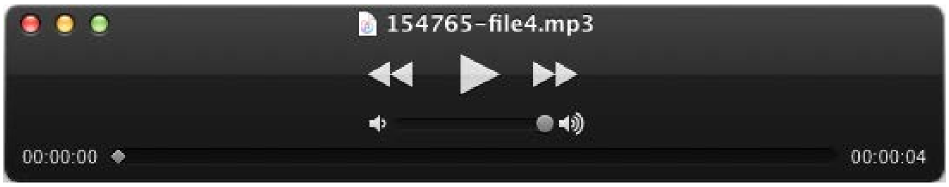

Supplemental Audio 3: VTC morph. Continuum stimuli between vocal tract characteristics (VTC) of monkey A and monkey B used in the experiment.
